# The Neurological Traces of Look-Alike Avatars

**DOI:** 10.3389/fnhum.2016.00392

**Published:** 2016-08-03

**Authors:** Mar Gonzalez-Franco, Anna I. Bellido, Kristopher J. Blom, Mel Slater, Antoni Rodriguez-Fornells

**Affiliations:** ^1^Microsoft ResearchRedmond, WA, USA; ^2^Experimental Virtual Environments for Neuroscience and Technology (EVENT) Laboratory, Department of Clinical Psychology and Psychobiology, University of BarcelonaBarcelona, Spain; ^3^Institute of Neuroscience, University of BarcelonaBarcelona, Spain; ^4^Catalan Institute for Research and Advanced Studies, ICREABarcelona, Spain; ^5^Department of Computer Science, University College LondonLondon, UK; ^6^Cognition and Brain Plasticity Group, Bellvitge Biomedical Research Institute—IDIBELL, L’Hospitalet de LlobregatBarcelona, Spain; ^7^Department of Basic Psychology, University of BarcelonaBarcelona, Spain

**Keywords:** self-recognition, face-recognition, visual cortex, event related potentials, memory, avatars

## Abstract

We designed an observational study where participants (*n* = 17) were exposed to pictures and look-alike avatars pictures of themselves, a familiar friend or an unfamiliar person. By measuring participants’ brain activity with electroencephalography (EEG), we found face-recognition event related potentials (ERPs) in the visual cortex, around 200–250 ms, to be prominent for the different familiarity levels. A less positive component was found for self-recognized pictures (P200) than pictures of others, showing similar effects in both real faces and look-alike avatars. A rapid adaptation in the same component was found when comparing the neural processing of avatar faces vs. real faces, as if avatars in general were assimilated as real face representations over time. ERP results also showed that in the case of the self-avatar, the P200 component correlated with more complex conscious encodings of self-representation, i.e., the difference in voltage in the P200 between the self-avatar and the self-picture was reduced in participants that felt the avatar looked like them. This study is put into context within the literature of self-recognition and face recognition in the visual cortex. Additionally, the implications of these results on look-alike avatars are discussed both for future virtual reality (VR) and neuroscience studies.

## Introduction

In the context of object observation and recognition, humans are particularly good at recognizing faces compared to other objects. Previous research has shown that the brain processing of faces differs from that of other objects (Axelrod et al., 2014). Several researchers have studied this particular effect using electroencephalography (EEG) (Bentin et al., 1996; Allison et al., 1999; Eimer, 2000; Caldara et al., 2003), MEG (Liu et al., 2002), and fMRI (Vuilleumier et al., 2001). In particular, EEG studies have associated different event related potential (ERP) N170 components in the visual cortex area during the observation of faces vs. other objects (Bentin et al., 1996; Allison et al., 1999; Axelrod et al., 2014). In healthy subjects faces elicit larger N170 potentials in the visual cortex than other non-face objects (e.g., cars, flowers; Bentin et al., 1996; Zion-Golumbic and Bentin, 2007). This suggests that the encoding of object categories can be signaled by the strength of the N170 (Eimer, 2011), showing a significant more negative voltage for faces just 170 ms after the stimulus onset. The expertise of participants with respect to the presented object seems to affect the ERPs: after a period of observational training of non-face objects the responses of N170 significantly decreased (20%) when compared to subjects who were untrained for those objects (Gauthier et al., 2003; Rossion et al., 2004). A similar training effect has been found for face processing in younger children who do not have the expertise of an adult; children of different ages show different brain potentials during face observation tasks (Taylor et al., 2004). However, the N170 component does not show evidence of face identification, or face memory access (Bentin and Deouell, 2000; Eimer, 2000; Tanaka et al., 2006). According to the literature, memory-related face processing first occurs 200 ms after the stimulus onset (Schweinberger et al., 2004; Kaufmann et al., 2009). Applying transcranial magnetic stimulation (TMS) in the same occipito-parietal part of the brain produced an impairment in face discrimination tasks on healthy participants (Rossion et al., 2003), similar to the deficiencies showed by patients with prosopagnosia who cannot recognize and identify faces (Pitcher et al., 2008, 2011).

In fact, these later brain mechanisms seem to play an important role not only in the distinction between faces and other objects, but also with respect to the evaluation of the emotional state of the face (Vuilleumier et al., 2001; Eimer and Holmes, 2007), and in the classification of faces with different degrees of familiarity (self, familiar, unfamiliar; Bentin and Deouell, 2000; Sui et al., 2006; Platek and Kemp, 2009; Keyes et al., 2010; Ramasubbu et al., 2011). When categorizing faces of different ages, races and genders, a larger P200 is found to outgroups (Ito and Urland, 2003; Ito and Bartholow, 2009; Tanaka and Pierce, 2009; Wiese, 2012); a positive P200 followed by negative N250 deflection has also been related to higher levels of familiarity of the face when observing self, familiar and unfamiliar faces (Tanaka et al., 2006; Kaufmann and Schweinberger, 2008; Wiese, 2012). In these components, self faces show significantly reduced voltage when compared to other people’s faces. Therefore, these mechanisms are sensitive to the owner of the face such that images of our own face are processed differently than the faces of others, i.e., familiarity of the face impacts its processing (Devue and Brédart, 2011). Thus, P200 has been proposed as the earliest component that indexes the stored face representations from the long-term memory, able of self-recognition (Pfütze et al., 2002). The aim of the present study was to investigate further these face recognition traces (Caharel et al., 2002; Tanaka et al., 2006; Kaufmann et al., 2009). In this context previous research with ERPs has found that caricaturing familiar and unfamiliar faces with distortions of up to 30% did not affect the processing of the familiar faces whereas the unfamiliar faces elicited significant effects (Kaufmann and Schweinberger, 2008). Furthermore, in that research the familiarity differences (familiar vs. unfamiliar) remained prominent both in veridical and caricature faces (Kaufmann and Schweinberger, 2008). Additional studies have shown tolerance in the processing of familiar faces even when they are distorted (Sandford and Burton, 2014).

In this article, we exploit previous research on self, familiar and unfamiliar faces to compare the ERP brain processing dynamics when participants are exposed to look-alike avatar faces vs. other avatars. Avatars are widely used in virtual reality (VR) to represent participants and other actors, and multiple behavioral studies have shown that participants responses can vary depending on the external appearance of the avatars (Peck et al., 2013), and whether the avatars looked like the participant (Streuber et al., 2009; Osimo et al., 2015). In this context, we aim to examine the extent to which avatar faces are processed as real faces, and also whether self-recognition on physically similar avatars may elicit measurable neurophysiological effects that relate to the actual subjective experience.

## Materials and Methods

### Participants

Seventeen neurologically healthy male subjects between the ages of 25 and 41 (M = 33, SD = 4.8), with normal or corrected vision participated in the experiment. Only males were used because of limitations on the pipeline to generate look-alike avatars. Participants were recruited via email from the laboratory mailing list. Since we wanted to study the effects of self-specific processing and familiarity, participants were matched with workmates and friends seen every day by the participants for the stimuli generation. Participants had never been exposed to the specific stimuli used in this experiment at the moment of recruiting and had not participated before on face observation studies. Subjects gave informed consent according to the Declaration of Helsinki, and the experiment was approved by the ethics committee of the Universitat de Barcelona.

### Look-Alike Avatars

**Figure 1:**
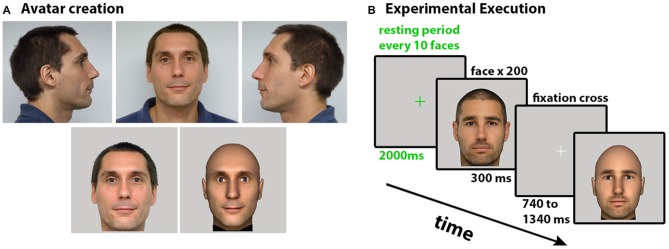
**(A)** Creation of the look-alike avatar, the three pictures used for the avatar generation on the top. On the bottom, the final pictures used for the experiment as real and virtual. **(B)** Experimental execution. The six faces (self-real, self-virtual, familiar-real, familiar-virtual, unfamiliar-real, unfamiliar-virtual) were randomly ordered in blocks of 10. Each face was displayed for 300 ms, followed by a variable time of 740–1340 ms in which a fixation cross appeared. After each block there was a short resting period of 2 s for blinking.

We took three photographs (front, left and right profile) of each participant in order to create their look-alike avatars. In all cases participants were requested to maintain a neutral facial expression to avoid emotion processing during the observation, since it has been shown that emotions on faces generate different brain traces and activate mirror neurons (Likowski et al., 2012). Avatars were generated in a matter of minutes using the fast creation of look-alike avatars pipeline described in Blom et al. (2014). The resultant avatars were tweaked manually to make minor smoothing and lighting improvements. The avatars were displayed without hair, focusing the experiment on the participant’s pure facial characteristics; none of the participants was actually bald. We used the original frontal picture and a frontal capture of the resulting avatar for the observation task (Figure 1A).

The real and the computer generated images were processed to equalize pupil-pupil distance across all, and to ensure that the vertical midline of the image bisected the face; this involved a minor scaling of the entire image, and left the normal proportions of the face invariant. The images were saved at 520 × 520 pixels, in color and with averaged luminance to avoid effects of uncontrolled interstimulus perceptual variance that have been found to influence the N170 component (Thierry et al., 2007). Controlling this particular variance seems to correct the N170 face sensitivity (Bentin et al., 2007).

### Stimuli

The experimental design included two factors: *Virtuality* and *Familiarity*. The *Virtuality* factor consisted of either a photographic image of a real person (Real) or an image of the look-alike avatar (Avatar) of that person. The *Familiarity* factor consisted of the three levels: self (Self), familiar (Familiar) or unfamiliar other (Unfamiliar). The person used for the *Unfamiliar* condition had no previous relation to any of the participants. Since we wanted to study the effects of self-specific processing rather than habituation to the face—e.g., participants see themselves in mirrors very frequently—the Familiar faces were extracted from friends seen every day by the participants.

In total, subjects were exposed to the six images, 200 times each, in a classical ERP setup resulting in a total of 1200 faces to be observed by each participant during the experiment that lasted approximately 30 min. Habituation to the stimuli was explored to test possible adaptation effects. The *Familiarity* factor was based on the existing literature on ERPs and self-face processing (Sui et al., 2006; Tanaka et al., 2006; Keyes et al., 2010).

The visual stimuli were implemented and displayed using the XVR programming system (Tecchia et al., 2010; Spanlang et al., 2014) on an Intel Core i7 at screen resolution 1920 × 1080 pixels. The six faces (*self-real, self-avatar, friend-real, friend-avatar, unfamiliar-real, unfamiliar-avatar*) were pseudo-randomly ordered in blocks of 10. Each face was displayed for 300 ms, followed by a variable time of 740–1340 ms in which a fixation cross appeared (Figure 1B). After each block of 10 pictures there was a resting period of 2 s for blinking. Participants were instructed to maintain the focus on the fixation point and to minimize blinks and eye movements.

### Questionnaire

Before mounting the EEG cap and starting the experiment we showed the faces one by one to the participants and administered a short questionnaire in which they had to rate the *realism* of both the real and avatar pictures from 1 (not alike at all) to 5 (looks completely like the real person). Specifically they were asked to answer whether: “*The picture looks like the real person*”. The realism of the faces when compared to the real person were not available for the unfamiliar case as participants did not know the real person. Instead they were asked to compare the similarity of the avatar face to the photographic picture face (from 1 to 5): “*The avatar face looks like the real face*”. This similarity rating was performed for all pairs of real-avatar pictures. In order to detect changes in the scoring that were due to the experimental exposure or adaptation effects, participants were asked to rate again all the faces and avatars after the experiment.

### EEG Recording

Continuous EEG was acquired from 64 active electrodes located at standard 10–20 positions with a g.HIamp multichannel amplifier manufactured by g.Tec Medical Engineering. Active ring electrodes (g.LADYbird) were used in a standardized cap (g.GAMMAcap), both from g.Tec. The activity was referenced to the earlobe and the ground electrode was located in the frontal area of the head. Signals where digitalized at 256 Hz frequency rate, a notch Butterworth filter 4th order from 48 to 52 Hz was used to eliminate the AC. Ocular movements were detected from FP1, FP2, AF7 and AF8.

The EEG was segmented offline into 1200 ms epochs starting 200 ms before the stimulus onset. Trials where the faces were consecutively repeated were rejected off-line. There was a repetition rate of 2.99%. Trials in which ocular movements were found (EOG greater than 50 μV) or the absolute amplitude of the signal at any electrode was greater than 150 μV were rejected. The average acceptance rate was of 75 ± 15% trials per participant.

ERPs time-locked to the onset of the stimuli for each condition and participant were averaged for epochs of −200 to 900 ms with the baseline set from −150 to 0 ms. Mean amplitudes were calculated where there were discernible peaks in the average ERP waveform for each of the 17 participants; these included the epochs N170 (160–220 ms) and P200 (250–300 ms) of the right and left occipito-parietal cortex, which are consistent with those proposed in previous self-recognition experiments (Sui et al., 2006; Tanaka et al., 2006; Keyes et al., 2010).

The original ERPs for each subject were also transformed into reference-free Current Source Density (CSD) estimates (μV/cm^2^ units) using a spherical spline surface Laplacian Matlab-based CSD toolbox (Kayser and Tenke, 2006) to better show the topographical maps.

## Results

### Evaluation of Faces

**Figure 2:**
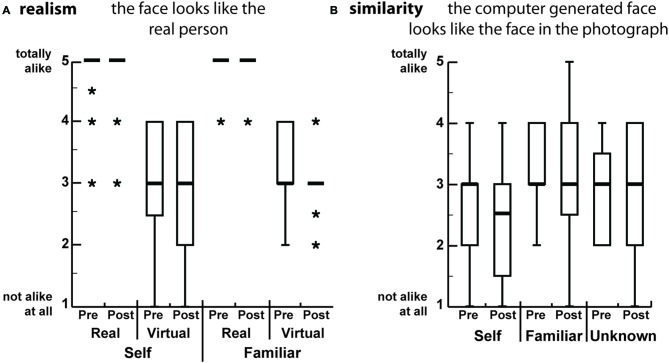
**Boxplots of the scores for the different faces, from the pre and post questionnaires.** The scores range from 1 (not alike at all) to 5 (totally looks alike). Participants rated the faces by **(A)** Realism: they compared the pictures to the real person that they knew; **(B)** Similarity: they compared the real picture to the picture of the avatar face independently of whether they knew not the person in the picture. The thick horizontal bars are the medians and the boxes are the interquartile ranges. The whiskers range from max (lower quartile − 1.5 * IQR, smallest value) to min (upper quartile + 1.5 * IQR, largest value). Values outside of this range are marked by *.

Participants rated of the *realism* of the faces by responding to the statement: “The face looks like the real person”; and the *similarity* between the avatar face and the photograph by responding to: “The avatar face looks like the face in the photograph” (Figure 2). After the experiment, participants were asked to rate again all the faces. There were no significant differences between any of the real or virtual faces in the pre-post scoring for the *realism* question (Wilcoxon signed-rank tests, all *p* > 0.45, *n* = 17), nor for the *similarity* pre-post (Wilcoxon signed-rank tests, all *p* > 0.55, *n* = 17). The real image scores served as a consistency control: none of the real pictures scored less than 4 out of the maximum of 5. Furthermore, there were no significant differences amongst the scores for all the avatar faces, which indicates that all of them were perceived similarly realistically (Friedman test, *p* > 0.37, *n* = 17).

There were significant differences when comparing the *realism* scores between the avatar and the real faces (Wilcoxon signed rank tests, all *p* < 0.001). This indicates that even though all the participants did recognize themselves and their confederates in the avatar faces, the avatars were not perceived as overly realistic as the real faces (Figure 2A). To the *similarity* question all avatars (self, familiar and unfamiliar) were rated as equally similar to their real counterparts (Figure 2B).

### EEG Analysis

**Figure 3:**
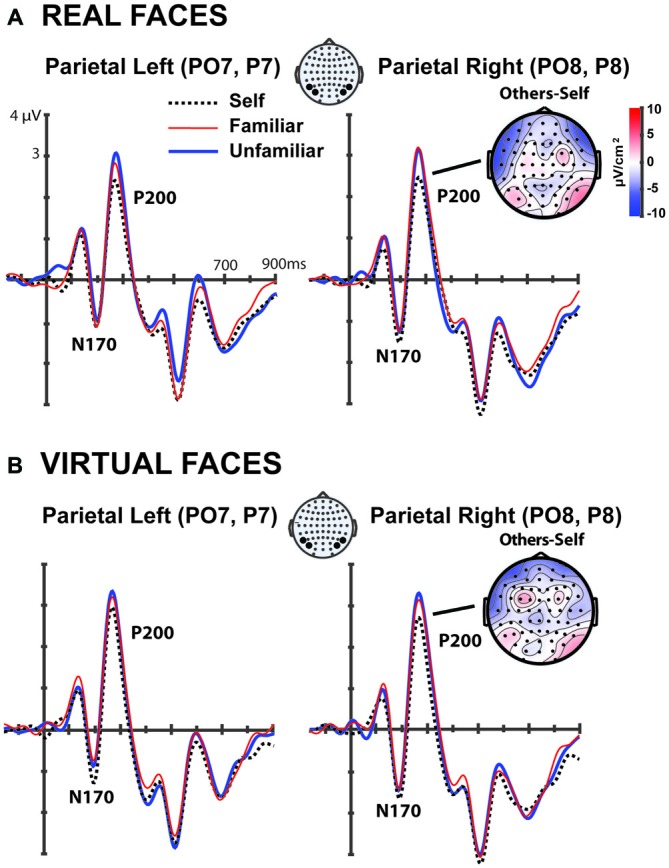
**Grand average event related potentials (ERPs) for the 17 subjects of the parietal electrodes (PO7, P7) and (PO8, P8) elicited during the first 100 trials by (A) Real faces: self, familiar, and other. (B)** Virtual faces: self, familiar, and unfamiliar. The current source density (CSD) topographical plots show how the difference between others-self for the P200 component is mainly located in the occipito-parietal cortex. A low pass filter (15 Hz, half-amplitude cut-off) was applied in these grand averaged graphs.

In order to explore how the faces were processed in the brain we proceeded to study well-defined visual processing-related ERP components in occipito-parietal cortex, such as the N170 and the P200 components (Bentin and Deouell, 2000; Caharel et al., 2002; Sui et al., 2006; Tanaka et al., 2006; Keyes et al., 2010). Visual inspection of Figure 3 shows a clear negative voltage for all the faces during the N170 (both for avatar and real faces); and a decreased voltage during the P200 and the N250 for the self-faces, both in the real and in the avatar conditions, as a result of familiarity processing (Tanaka et al., 2006).

The amplitude of each component was extracted for each participant in the specified time-windows (N170: 160–220 ms; P200: 250–300 ms) and analyzed via a repeated measures ANOVA with three within-subject factors: *Hemisphere* (left (PO7, P7), right (PO8, P8)) × *Familiarity* (self, familiar, other) × *Virtuality* (real, avatar). Importantly, only the first 100 trials of each condition are taken into account for this section to avoid the effects of adaptation (see below for the analysis of fast adaptation). The Mauchly test was run to account for significant differences on the variances. In cases where the test was positive (<0.05) we applied and noted the corresponding correction for sphericity.

#### N170

No significant main effects were found for the N170; there was a non-significant trend for *Familiarity* (*F*_(2,32)_ = 2.916, *p* = 0.069) and an interaction between *Hemisphere* × *Virtuality* (*F*_(1,16)_ = 5.076, *p* = 0.039). In a *post hoc* analysis only of the *virtual* faces, a trend was found for *Familiarity* only in the *left hemisphere* (*F*_(2,32)_ = 2.552, *p* < 0.094), however no significant effects were found on the r*eal* faces. No differences were found between the real and the virtual faces. Meaning that all faces, both virtual and real, were indeed classified as the same category in the N170 component. This is in agreement with the literature that has reported the N170 to be able to differentiate object categories, being so that observation of faces produces a more negative voltage than observation of other objects (Bentin et al., 1996; Zion-Golumbic and Bentin, 2007).

#### P200

The same repeated measures ANOVA was run on the P200 component. This analysis showed a significant main effect of *Familiarity* (*F*_(2,32)_ = 7.253, *p* < 0.003; Figure 3). *Post hoc* pairwise comparisons pooling together the amplitude in all occipito-parietal electrode locations (PO7, P7, PO8, P8) for both hemispheres showed significant differences between *self* (2.681 ± SE 0.319 μv) vs. *familiar* (3.147 ± SE 0.298 μv; *t* = 2.712, *p* = 0.015, *df* = 16) and *self* vs. *unfamiliar* (3.277 ± SE 0.298 μv; *t* = 3.403, *p* = 0.004, *df* = 16) but not between *familiar* vs. *unfamiliar* (*t* = 0.9, *p* = 0.381, *df* = 16), indicating the existence of a self-oriented encoding process (Figure 3).

**Figure 4:**
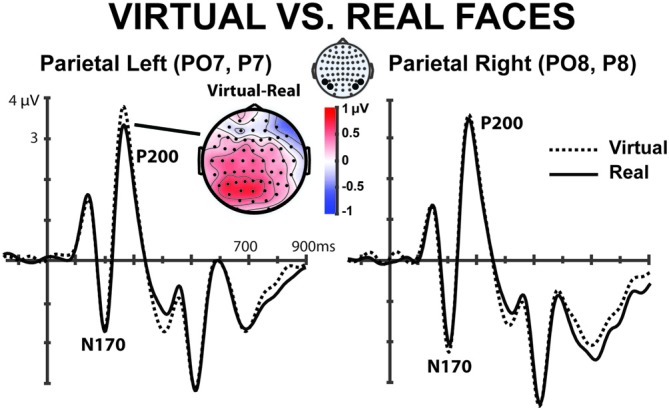
**Grand average ERPs of the 17 subjects in the parietal electrodes (PO7, P7) and (PO8, P8) elicited by the real and the computer generated faces for the first 100 trials.** There is a significant difference in the P200, mainly in the parietal left electrodes. A low pass filter (15 Hz, half-amplitude cut-off) was applied in these grand averaged graphs.

No interactions were found between *Familiarity* ×*Virtuality*, and in fact, both the avatar and the real faces did show similar *Familiarity* processing. More precisely when analyzing separately the *avatar* from the *real* faces we find that: the *self-avatar* (2.453 ± SE 0.293 μv) was processed with a significantly reduced voltage than the *familiar-avatar* (2.844 ± SE 0.265 μv) and the *unfamiliar-avatar* (2.953 ± SE 0.282 μv; *t* = 3.984, *p* < 0.033, *df* = 16). Similar effects were also found for the *self-real* (2.147 ± SE 0.318 μv) vs. the *familiar-real* (2.688 ± SE 0.306 μv) and vs. the *unfamiliar-real* (2.727 ± SE 0.343 μv; *t* = 2.407, *p* < 0.05, *df* = 16). In contrast, the pairwise comparison *familiar* vs. *unfamiliar* was not significant for any cases (*t* < 0.8, *p* > 0.3, *df* = 16), which can be explained if the neural mechanisms related to the P200 component were oriented mainly towards self-recognition and not so much towards the recognition of others’ faces (Figure 4). These effects seem to be stronger in the *right hemisphere* than the *left hemisphere*, although no interaction was found between *Familiarity* × *Hemisphere* visual inspection (Figure 3) seems to shows stronger effects on the right hemisphere.

Interestingly, regarding the nature of the face presented, we find a significant within subjects main effect for *Virtuality* (*F*_(1,16)_ = 7.946, *p* = 0.012) as well as an interaction between *Hemisphere* × *Virtuality* (*F*_(1,16)_ = 4.335, *p* = 0.05). *Avatar faces* (3.212 ± SE 0.249 μv) elicited larger amplitudes when compared to *real faces* (2.816 ± SE 0.360 μv) in the *left hemisphere* (*t* = 4.430, *p* < 0.001, *df* = 16). The same effect was not found in the *right hemisphere* locations (*t* = 0.722, *p* = 0.480, *df* = 16). This clear difference between virtual and real faces in the P200 component is depicted in Figure 4.

When observing the particular case of *self-real vs. self-avatar* we found that in the *left hemisphere* the processing was significantly different for both types of faces (*t* = 2.987, *p* = 0.009, *df* = 16, *self-avatar* = 2.56 ± SE 0.35 μv, *self-real* = 2.11 ± SE 0.35 μv), while the same did not occur in the right hemisphere (*t* = 0.763, *p* = 0.457, *df* = 16, *self-avatar* = 2.33 ± SE 0.28 μv, *self-real* = 2.18 ± SE 0.33 μv). These results suggest that while a self-recognition effect is mostly present in the *right hemisphere* where the *self-avatar* was classified in a similar way to the *self-real*, a *Virtuality* effect is found on the left hemisphere as if the *avatar-faces* were being dissociated from the *real-faces*.

Overall these results suggest that the amplitude of the P200 component is sensitive to the nature of the face, whether it is a computer generated avatar or a real face, as well as the *familiarity* level of the face, and more precisely the P200 seems to be oriented towards self-recognition in faces. In agreement with previous findings (Bentin et al., 1996; Zion-Golumbic and Bentin, 2007), this component might be related to the neural processes involved in distinguishing self from other faces. This particular effect of self-identification and familiarity seems to work for both the *avatar* and for the *real* faces, indicating that avatars that look alike the self are recognized as the self to a higher degree than the other avatars. Despite there is an evident visual difference between the computer generated avatars in this experiment and the real pictures, the fact that non-significant differences are found in ERPs traces is in agreement with previous findings showing large tolerance to distortions in familiar faces during self-recognition (Kaufmann and Schweinberger, 2008; Sandford and Burton, 2014).

### Rapid Adaptation Effects

**Figure 5:**
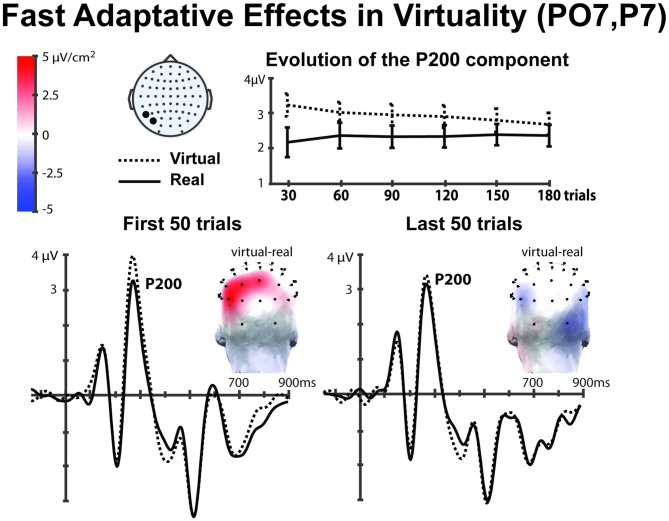
**The top panel shows the time evolution of the P200 amplitude in the left tempo-parietal cortex (P07, P7); presenting the cumulative voltage over blocks of 30 trials (the error bars show the standard error of the different participants ERPs).** We observe how in the first trials the virtual and the real faces are processed as different objects; however, this effect is reduced after the overexposure. In the panel below we observe the grand averaged ERPs of the first and last 50 trials, and a clear reduction of the P200 component is also observed. The topographical plots show the CSD of the P200 component difference (avatar-real) in the scalp, we can see how the difference decreases over the last trials. A low pass filter (15 Hz, half-amplitude cut-off) was applied to these grand averaged graphs.

In the previous section, we found the P200 component to be sensitive to both *Virtuality* and* Familiarity*. In this section, we investigate further the modulation of these effects over time. In Figure 5 we can observe the evolution of the amplitude of the P200 component for the *Virtuality* parameter across the whole experiment. The amplitude of the P200 component was computed in bins of 30 trials for both *avatar* and *real* faces (pooling together all the conditions) in the left hemisphere, where the effect of *Virtuality* was stronger (see previous section). An important observation in this Figure 5 (top panel) is the clear tendency of the voltage amplitude of the P200 component to progressively reduce over time in the avatar condition, tending to merge with the real face condition. Figure 5 (lower panel) also shows the grand average ERP for the first 50 and last 50 trials of each condition showing a clear reduction of the P200 amplitude differences in the last trials for both types of real and avatar faces.

In order to analyze these effects, we conducted a repeated measures ANOVA with factors *Time* (bins, 1–6) × *Virtuality* (real, avatar) focused on occipito-parietal left-hemisphere electrodes (PO7, P7) where the effect was observed to be larger (Figure 4). This analysis showed a significant main effect on *Virtuality* (*F*_(1,16)_ = 18.407, *p* = 0.001). Importantly and coherently with Figure 5 (top), a significant *time* interaction effect was found (*Time* × *Virtuality* (*F*_(5,80)_ = 2.865, *p* = 0.02), indicating a decay of the P200 amplitude over time for the avatar faces. Further pairwise comparisons showed that while a significant *Virtuality* effect was present in the first 50 trials (*t* = 4.037, *p* = 0.001, *df* = 16), no significant effects were observed in the last 50 trials (*t* = 1.169, *p* = 0.26, *df* = 16).

These findings suggest that the avatar faces are initially processed as different from the real faces, as if they were not faces but another kind of object, which is analogous to what happens with exposure to faces vs. flowers or other objects (Bentin et al., 1996; Allison et al., 1999; Keyes et al., 2010). Nevertheless, this effect is reduced over time, and both real and avatar faces eventually converge to be classified as the same class of objects: faces.

A similar analysis was run to study *Familiarity* effects over time at the P200 component (combining both right and left hemisphere). For this purpose, bins of 30 trials were used in the repeated measures ANOVA with factors *Time* (bins, 1–6) × *Familiarity* (self, familiar, other). A significant main effect was found for *Familiarity* (*F*_(2,32)_ = 5.308, *p* = 0.010); however, no interaction effects were observed for *Time* × *Familiarity* on the P200 component (*F*_(10,160)_ < 1). These results suggest that the effect of *familiarity* did not significantly decay over time in the present design. The analysis was also run with the avatar faces alone and there was no interaction of *Time.* This indicated that the self-recognition mechanisms are still functioning after longer exposures and both the self-avatar and the self-real faces are processed as belonging to one familiarity level, which is different to the other faces.

### Self-Identification Subjective Scores Match the Neurphysiological Responses

In the previous sections we have shown that the P200 amplitude was dependent on the *Familiarity* factor, and more precisely to the self-recognition in both real and avatar faces. In this section, we explore whether neurophysiological voltage could be related to higher cognitive processes such as the subjective self-identification score.

Since different participants may have different amplitudes in their components, we normalized the amplitude by calculating the voltage difference from the *self-avatar* to the *self-real* face for each participant (*self-avatar—self-real*) pooling together all the occipito-parietal electrodes in both hemispheres (PO7, PO8, P7, P8). A zero value coming out from that difference would indicate an equal voltage for the real picture and for the avatar, thus a greater self-identification, while more positive values would mean a lesser self-identification with the avatars, since in general a more reduced voltage in the P200 is associated with self-identification (Keyes et al., 2010).

**Figure 6:**
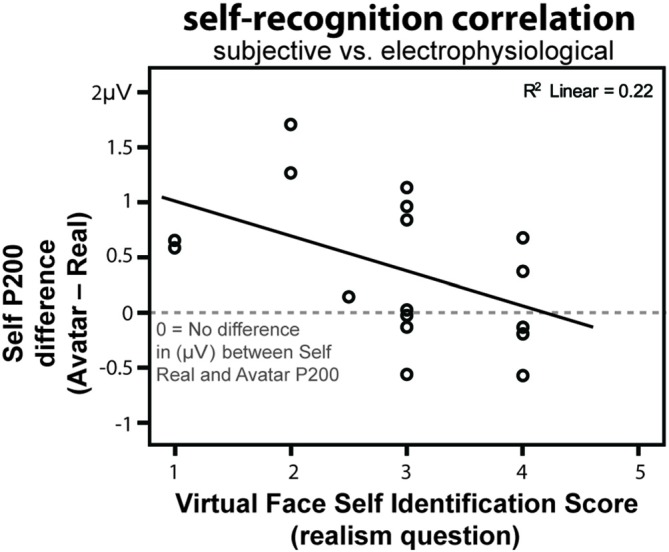
**Correlation between the virtual self-face identification scores in the realism question and the P200 voltage difference between *self-avatar–self-real*.** The closer the voltage difference is to zero the greater the identification.

The resulting voltage difference was then contrasted to the initial reported score of *realism* for the self-avatar face. In that question participants rated whether the avatar looked like themselves. A significant Spearman Correlation was found between the difference voltage (*self-avatar—self-real*) and the subjective score (*r* = −0.47, *n* = 17, *p* = 0.045; Figure 6).

## Discussion

Avatars have been used successfully as feasible substitutes of the body to study different aspects of neuroscience and psychology (Banakou et al., 2013; Banakou and Slater, 2014; González-Franco et al., 2014; Padrao et al., 2016). Some studies have found that behavioral responses may be modulated when such avatars look like the participants, indicating that there are some self-identification effects related to embodiment (Aymerich-Franch et al., 2012; Fox et al., 2012; Osimo et al., 2015). The current study presents novel neural evidence related to the process of self-identification in avatars. Differences in ERPs were prominent for the *Familiarity* levels of both real and avatar faces, more in particular a significant reduced voltage in the P200 component at the occipito-parietal locations was found both for the self-avatar and the self-real face conditions, showing that the look-alike avatars were self-recognized to a higher extent than other avatars. This P200 voltage response has been previously described in other self-recognition experiments (Caharel et al., 2002; Tanaka et al., 2006; Keyes et al., 2010). Despite the evident visual difference between the computer generated avatars in this experiment and the real faces (Figure 1), the fact that non-significant differences are found in the P200 traces for the self-avatar vs. the self-real is in agreement with previous findings: caricaturing familiar faces with distortions of up to 30% did not affect the neural processing of P200 or N250 (Kaufmann and Schweinberger, 2008), nor did the observation of low quality CCTV images (Bruce et al., 1999). Furthermore, in Kaufmann and Schweinberger (2008) the familiarity differences (familiar vs. unfamiliar) remained prominent both in veridical and caricature faces, which is also coherent with our results that find the P200 effects of self-recognition to be prominent both in real as well as in avatar faces. Our results therefore also contributed to confirm the tolerance in the processing of self-faces even when they are distorted (Sandford and Burton, 2014).

In our experiment a significant correlation was found between the self-identification scoring and the P200 component related with self-recognition mechanisms: the more the participants scored the self-avatar face to be like the real face, the smaller the difference in voltage between the self-avatar and self-real faces. Hence, the self-avatar face seems to have been processed at a low level (ERPs) in congruence with higher-cognitive functions (subjective questionnaire). The correlation between the subjective scoring and the self-identification mechanism of the P200 suggests that, either the conscious self-reporting is somehow influenced by the underlying visual processing of the faces, or the other way around. Anyhow given that both higher and lower cognitive functions are aligned, this correlation can be interpreted also as a validation of the P200 being indeed related to self-recognition. Thus providing yet more evidence to existing studies by other authors (Caharel et al., 2002; Tanaka et al., 2006; Kaufmann and Schweinberger, 2008; Keyes et al., 2010).

Besides, our results showed that all faces, both avatars and real faces, were indeed classified as the same object category in the N170 component where no significant voltage differences were found. The N170 component has been widely regarded as a neural trace of object categorization processing, being so that observation of faces produces a more negative voltage than observation of other objects such as flowers or cars (Bentin et al., 1996; Zion-Golumbic and Bentin, 2007). In our experiment both real and avatar faces did not exhibit strong differences in the N170 component. However, the amplitude of the P200 component was sensitive to the nature of the face for the earliest trials, i.e., whether the face was computer generated or real. Indeed, we found evidence in the left ocipito-parietal locations that the *Virtuality* of the faces affected the voltage of the P200 component only in the first trials of the experiment, but not in later trials where the effect disappears and both avatar and real faces are processed the same (Figure 5). This result seems to indicate that both computer generated and real faces elicited equivalent ERP components after a fast adaptation process. Visual expertise effect has been described before during observation of faces with different familiarity and it has been related the P200 amplitude to the activation of pre-existing and acquired face representations (Tanaka et al., 2006), similar effects have also been associated to memory related face observation studies (Ito and Urland, 2003; Schweinberger et al., 2004; Ito and Bartholow, 2009). Furthermore, studies with children of different ages also showed how expertise influences face processing (Pfütze et al., 2002; Taylor et al., 2004). We suggest that in the present study participants underwent a similar adaptation: since they were not previously exposed to the avatar faces they required a certain amount of visual exposure (repetition) to gain visual expertise on these new faces.

Overall, the present study contributes towards expanding the frontiers of self-recognition and face processing. More precisely, we provide further evidence of the tolerance of the brain in regards to self-recognition and external appearance distortions, as well as of the link between higher level functions and the internal low level processing of faces during self-recognition. Future research on this area could help explore the link between the neurophysiological signatures and the subjective rejection of faces in complex scenarios or contexts, such as the uncanny valley effect (Seyama and Nagayama, 2007), where very realistic computer generated faces produce a drastic emotional rejection by the observers (Cheetham et al., 2011).

We provide objective evidence that avatar faces are interpreted just as any other face and that look-alike avatars can be recognized as the self to a greater extent than other avatars, and thus be processed similarly to the real self. In general, this finding has important implications for future and existing research as it validates previous studies that have shown a change in the participant’s behavior when avatars bear external physical similarities to themselves (Streuber et al., 2009; Fox et al., 2012; Osimo et al., 2015). With ERPs not only can we study the effects of look-alike computer generated avatars, but also track on-line adaptive changes without requesting participants to continuously provide behavioral judgments. Future research in this field could explore whether the neurophysiological response generated over a random avatar face can be modulated by a virtual body ownership experience, i.e., if after being embodied in an avatar, its face is perceived as closer to the self-face than before. In this way the methodology presented may also provide a new objective measure to evaluate the appearance effects across time on people whose body has been substituted in VR. This could be a useful tool to explore “other-race-effects” of ERPs (Balas and Nelson, 2010) and racial bias modulation after exposed to other race avatars (Peck et al., 2013).

## Significance Statement

This article presents novel findings in the field of self-recognition. It shows evidence of face-memory accessing for avatar faces that corresponds to self-recognition in computer generated look-alike avatars. Furthermore, the neural traces for self-recognition are correlated to the subjective feeling of the avatar looking like the participant. These findings predict that avatars that look like the participants will have stronger self-representation impact than arbitrarily chosen avatars.

## Author Contributions

MG-F and KJB conceived and designed the work; MG-F and AIB did the implementation and run the experiments. MG-F and AR-F did the analysis, and interpretation of data. MG-F, MS and AR-F wrote the article.

## Conflict of Interest Statement

The authors declare that the research was conducted in the absence of any commercial or financial relationships that could be construed as a potential conflict of interest.
